# STBase: One Million Species Trees for Comparative Biology

**DOI:** 10.1371/journal.pone.0117987

**Published:** 2015-02-13

**Authors:** Michelle M. McMahon, Akshay Deepak, David Fernández-Baca, Darren Boss, Michael J. Sanderson

**Affiliations:** 1 School of Plant Sciences, University of Arizona, Tucson, AZ, 85721, United States of America; 2 Department of Computer Science, Iowa State University, Ames, IA, 50011, United States of America; 3 Department of Ecology and Evolutionary Biology, University of Arizona, Tucson, AZ, 85721, United States of America; Université Paris-Sud, FRANCE

## Abstract

Comprehensively sampled phylogenetic trees provide the most compelling foundations for strong inferences in comparative evolutionary biology. Mismatches are common, however, between the taxa for which comparative data are available and the taxa sampled by published phylogenetic analyses. Moreover, many published phylogenies are gene trees, which cannot always be adapted immediately for species level comparisons because of discordance, gene duplication, and other confounding biological processes. A new database, STBase, lets comparative biologists quickly retrieve species level phylogenetic hypotheses in response to a query list of species names. The database consists of 1 million single- and multi-locus data sets, each with a confidence set of 1000 putative species trees, computed from GenBank sequence data for 413,000 eukaryotic taxa. Two bodies of theoretical work are leveraged to aid in the assembly of multi-locus concatenated data sets for species tree construction. First, multiply labeled gene trees are pruned to conflict-free singly-labeled species-level trees that can be combined between loci. Second, impacts of missing data in multi-locus data sets are ameliorated by assembling only decisive data sets. Data sets overlapping with the user’s query are ranked using a scheme that depends on user-provided weights for tree quality and for taxonomic overlap of the tree with the query. Retrieval times are independent of the size of the database, typically a few seconds. Tree quality is assessed by a real-time evaluation of bootstrap support on just the overlapping subtree. Associated sequence alignments, tree files and metadata can be downloaded for subsequent analysis. STBase provides a tool for comparative biologists interested in exploiting the most relevant sequence data available for the taxa of interest. It may also serve as a prototype for future species tree oriented databases and as a resource for assembly of larger species phylogenies from precomputed trees.

## Introduction

Phylogenetic trees have greatly altered comparative biology by rearranging the context for comparison, enhancing statistical power of comparative tests, and broadening taxonomic scope [1, 2]. In recent years the demand for phylogenetic trees has been so high that comparative biologists themselves have frequently turned to heuristic or even non-algorithmic methods for assembling trees comprehensive enough to contain the taxa in which they are interested (e.g., Pringle et al.’s [3] use of the Phylomatic Project [4]). This reflects one basic impediment to phylogenetic comparative studies: the mismatch between the set of taxa present in published or databased phylogenetic trees and the set of taxa for which comparative data are available. For example, the Royal Botanic Gardens, Kew, maintains a database of morphological and biochemical data on seeds of angiosperms [5], which has been used in comparative analyses such as Moles et al.’s [6] study of the correlates of seed size variation. Currently, of the 2,572 species in the angiosperm eudicot clade that have data for the trait “percent oil content,” only some 64% have sequences in GenBank, even though eudicots are arguably one of the best sampled species-rich taxonomic groups in the tree of life (the overall species level sequence coverage across described eukaryotes is closer to 10% [7]). Moreover, the eudicots that are represented in GenBank are not all sequenced for the same set of homologous loci; instead, taxon coverage is patchy among various loci, so that phylogenetic trees assembled from GenBank sequence are more limited in their taxon coverage than the count of species in GenBank suggests.

One strategy to overcome this mismatch is assembly of ultra-large, dense phylogenies of particular clades [8–14], or particular regions of the world [15–18], depending on the biological question. However, scaling up phylogenetic inference presents numerous computational challenges [19–22], especially in handling the patchy coverage across multiple sparsely sampled loci [23–25]. An alternative strategy, which should be useful in the near term, is to assemble a very large collection of phylogenetic trees of small to medium scale, and optimize the delivery of these trees via efficient search and retrieval. This is the strategy we have employed here. Mismatch is reduced by using available sequence data to compute new trees, so users are not limited to taxon sets from individual published data sets. As larger trees are needed, data sets and/or trees can be pieced together by other algorithms (see Discussion). One clear advantage of this is that it allows relatively robust estimation of reliability (yet another computational problem that does not scale well), and these estimates of reliability can be returned to the user.

In addition to the frequent mismatch between taxon sets of interest and taxon sets that are in published trees, a second basic impediment to harnessing available phylogenetic trees in comparative biology is that many are gene trees. More generally, many are “multrees”, that is, trees having multiple sequences with the same taxon name. This can arise because of multiple sampling of individuals within a species, multiple alleles at the same locus, or multiple paralogs in the same gene family. Several tree databases implicitly allow such trees, including TreeBASE [26] and the PhyLoTA database [27], in addition to genomic databases that literally set out to archive gene trees instead of species trees (e.g., PFAM [28], TreeFam [29], PhylomeDB [30], and within Ensembl [31]). However, it is not straightforward to undertake comparative biology of structure, function, ecology, etc., using multrees, especially those riddled with gene duplications, losses, or lateral transfer. The construction of species trees from gene trees is an active area of research, with an extensive and long-standing literature [32–36]. We take an extremely conservative view of the problem, and implement a method [37] to ameliorate this impediment, which we hope will at least expose some of the problems that must be resolved in future database efforts.

In this paper we describe a new database of precomputed phylogenetic trees of eukaryotes, STBase (“Species Tree Database”), optimized for use by comparative biologists. In it we deposit one billion pre-computed phylogenies built from one million single- and multi-locus datasets assembled from GenBank. Selection of taxa and loci for data set assembly is guided by recent theory on optimal multigene data set construction [23, 24] and treatment of multrees [37]. We join this with a scalable search engine that accepts lists of taxon names (genera, species, or subspecific) and efficiently returns a ranked list of trees, the subtrees that overlap with the taxa of interest, and support values.

## Construction and Content

### Overview

The goal of STBase is to provide a tool that accepts a user’s query list of taxon names and returns a ranked list of good “hits” to a database of phylogenetic trees. A “hit,” meant to be analogous to BLAST searches [38], occurs when the search engine finds a data set that contains a minimum number of the query taxa. STBase does not accommodate fuzzy searches at this time; taxon names must be spelled the same as in GenBank taxonomy. Each “hit” has an associated set of 1000 trees created by bootstrap analysis. Subtrees, with confidence estimates, are created by pruning each bootstrap tree to the taxa of interest; the majority rule consensus of these bootstrap subtrees is then returned to the user. To quantify what “good hit” means, we construct a scoring function that increases with the quality of the tree and the amount of taxonomic overlap between the tree and the query. We assume that tree quality can be characterized by including a confidence set of trees in the database, computed, for example, by bootstrapping (as here) or by sampling the posterior distribution [1]. Let A be the query list, and *h* be a user-supplied preference indicating the relative importance of tree quality vs. taxon overlap. For any tree, T, let L(T) be the taxa in the tree, T | A be the subtree restricted to just the query taxa, and L(T | A) be the taxa shared between the query and the tree. Then define *w* (L(T | A)) to be an increasing function of this overlap. Let q (T | A) be some increasing function of the quality of the subtree. The score of a “hit” on (precomputed) tree T for query list A is then

S=w(L(T|A))+h×q(T|A).

Defining the score in this way allows the user to seek trees that overlap more extensively with their query list of taxa (as the scalar *h* approaches 0) or to prefer subtrees that have high quality as indicated by their bootstrap values (as *h* increases). On average, we expect larger trees to be less well supported [39]; intermediate values of *h* will return trees that may present a compromise between the two extremes of larger trees vs. better trees. The quality score, q (T | A), is calculated by multiplying the average bootstrap support (for nodes above 50%) by the proportion of resolved nodes in the majority rule consensus tree. The overlap function, *w* (L(T | A)), is the number of overlapping taxa divided by the number of query taxa that are in the database (rather than the larger set of query taxa that might include taxa not found in GenBank at all). To ensure the score is comparable and therefore useful in ranking the results, we normalize the score to range from 0 to 100 by multiplying the overlap function by 100 and by dividing the result by 1+ *h* (*h* is a positive number; the user interface has a slider bar allowing selection between 0.01 and 10.0 with a default value of 1.0). Consider, for example, a user-supplied input list of 200 taxon names, all of which are found in GenBank (note that taxon names missing from GenBank do not affect the ranking of the results). Suppose the database contains a large tree of 1200 taxa that shares 80 of the names on the query list, that the majority rule consensus tree (MRT) of 1000 bootstrapped trees, pruned to those 80 taxa (see below for details on pruning), is fully resolved and has an average bootstrap value of 70%, and the user has selected an *h* value of 0.5. The normalized score for this tree: S = ((80/200) × 100 + 0.5 × 70) / (1 + 0.5) = 50. Even though thousands of trees may be returned from any given query, their scores are calculated on the fly, so that as the user changes *h*, the ranking of trees is adjusted immediately.

### Tree Construction

**Fig 1:**
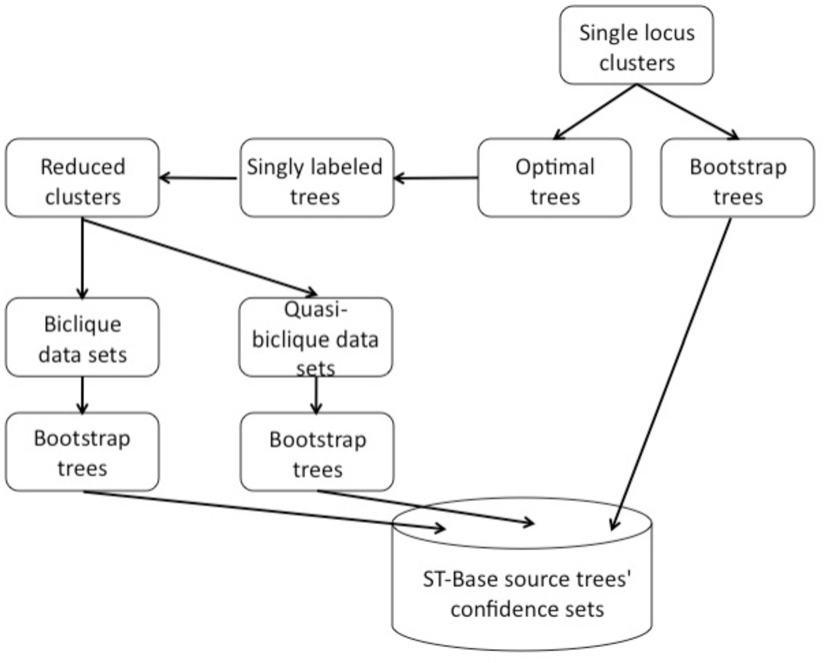
Pipeline for tree construction. Single-locus clusters are assembled from GenBank nucleotide data following procedures in Phylota [27]. One thousand fast parsimony bootstrap trees are reconstructed and stored in the database. Maximum likelihood trees are reconstructed and are used to guide sequence selection for the singly-labeled trees (see text for full description). Reduced clusters are assembled into various multi-locus data sets, each of which results in one thousand bootstrap trees, deposited in the database.

*Single-locus data sets*. Fig. 1 illustrates our tree construction pipeline. Single-locus nucleotide data sets (Table 1) were assembled from GenBank rel. 184 largely according to the PhyLoTA pipeline described elsewhere [27]. Briefly, data sets were constructed from sequences within size-limited eukaryotic taxonomic groups (“hub groups”). Each hub group was selected such that the total number of sequences from all of its members would not exceed 35,000 (excluding model organisms; cf. [27] for details). Membership in the group was determined by the NCBI taxonomy. This approach resulted in a set of 517 taxonomic groups that corresponded in practice very roughly to the rank of Linnean orders. Within each hub group, clusters of homologous sequences were identified by all-against-all BLAST searches and single-linkage clustering using 50% minimal overlap requirements. This operation was then repeated for each descendant group of the hub group in the NCBI hierarchy, inducing a set of parent-child relationships among clusters. From an original pool of 5,798,234 sequences among 413,628 distinct taxa, a set of 343,888 taxa was retained in 160,801 phylogenetically informative clusters (i.e., clusters with four or more taxa). The largest cluster has 20,125 sequences, the mean cluster size is 69.8, and there are 133 clusters with ≥ 5,000 sequences.

**Table 1:** Summary statistics for the three kinds of data sets.

	Number of data sets	Loci (mean and range)	Taxa^1^ (mean and range)	Data set size^2^ (mean and range)	Mean support (fraction of resolved nodes on MRT)
Single-locus clusters	160,801^3^	1 (1–1)	63.1 (4–8767)	63.1 (4–8767)	0.51
Bicliques	762,529	9.8 (2–91)	15.6 (4–510)	142.3 (8–1526)	0.84
Decisive quasi-bicliques	67,103	12.4 (2–386)	27.8 (5–1406) ^4^	234.7 (10–9516)	0.68
Total database	990,433	8.5 (1–386)	24.1 (4–8767)	135.7 (4–9516)	0.79

^1^We require a minimum of four taxa in a data set, required for potentially informative relationships in an unrooted tree.

^2^Product of number of loci and number of taxa.

^3^Of these, 111,433 were multrees. Some 11,358 data sets had fewer than 4 taxa after multree reduction, so only 149,443 were used to build multi-locus data sets.

^4^Because we require four taxa for minimal potential phylogenetic informativeness, a decisive quasi-biclique data set, which has some entries missing, must have a minimum of five taxa (else it would be a biclique, proper).

**Fig 2:**
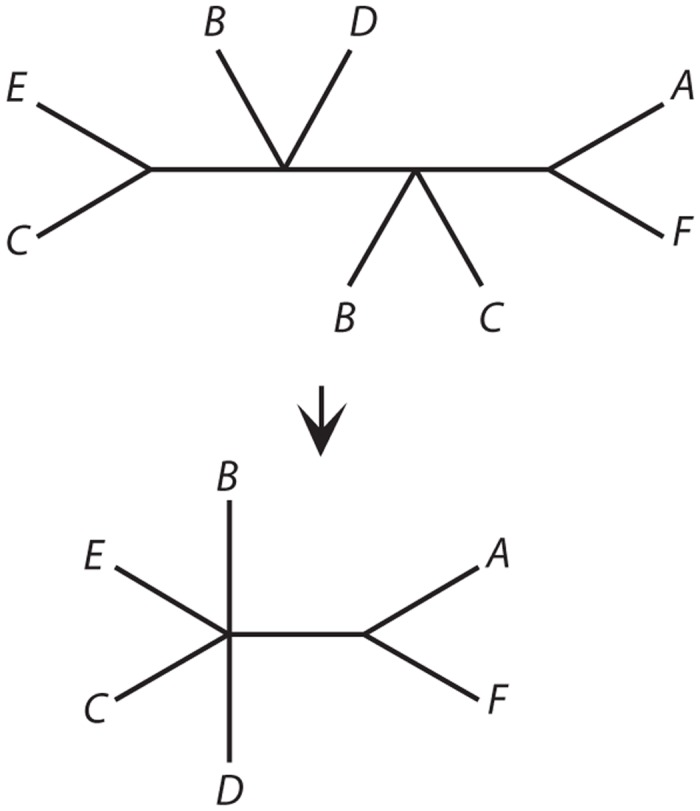
Illustration of the multree reduction algorithm [37]. The upper tree is a multree, i.e., it has at least one label that is found on more than one terminal. Furthermore, it displays quartets (subtrees on four taxa) that are in conflict: *BD*|*EC* conflicts with *BC*|*DE*. The reduced form of the tree, below, is a singly-labeled tree. This is a conservative statement about species relationships in the sense that it eliminates conflict (while introducing no new information).

**Fig 3:**
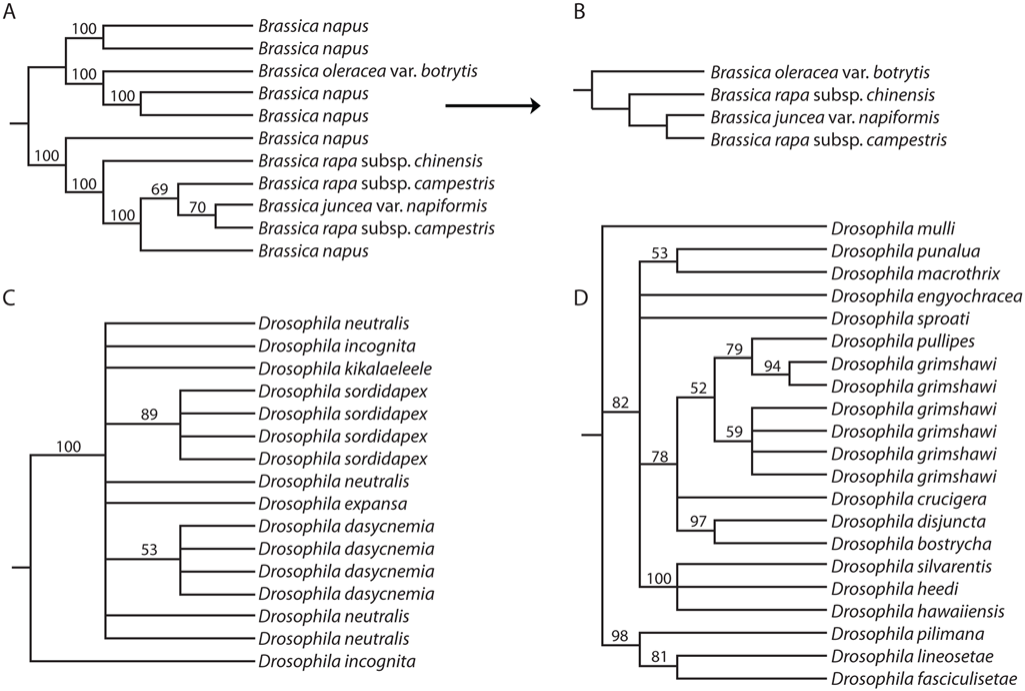
Examples of multree reduction in single locus data sets. A. Highly ranked tree for query “*Brassica*” inferred from a single-locus data set aligned at the level of Brassicaceae (data set #56065; phenylalanine ammonia-lyase). B. Reduction of the tree in A to species-level relationships without conflict. Note the loss of one taxon, *Brassica napus*; this taxon was present in many quartets in the original tree, but each one was contradicted by at least one other quartet. Therefore no conflict-free information was present for this taxon, and it was removed by the algorithm. C. Low ranked tree from query “*Drosophila*” (data set #130188; SMOX gene) in which there are no informative edges so the multree reduction produces a null result (no edges, no taxa; not shown). D. Intermediate ranked tree in which only one taxon has multiple sequences and the reduced singly-labeled tree contains all taxa (data set #91190; ‘yolk protein 1’; reduced tree not shown, but can be obtained by deleting all but one of the leaves labeled *Drosophila grimshawi*).

Many (69%) of these clusters included at least one taxon ID multiple times; such taxonomically redundant sequences could be due to sampling of multiple individuals, or they could represent multiple alleles or even paralogous loci. Taxon names occurring more than once in a data set can be referred to as “multaxa”, and the trees from such data sets are “multrees” [40]. We exploited a recently described multree reduction algorithm [37] to extract from each of these multrees a singly labeled “reduced” tree that is guaranteed to retain the maximum amount of conflict-free species-level information (Fig. 2). In brief, the algorithm evaluates quartets (an edge, or branch, separating two pairs of taxa), and finds those that are not in conflict with other quartets on the same set of taxa. The effect of the algorithm is to remove conflicted edges and any taxa that participate in no conflict-free quartets (Figs. 2, 3; see [37] for formal description). This is a conservative procedure that limits the number of false positive species relationships. Importantly, it is robust to the biological reasons for the presence of multiple sequences. They may arise through gene duplication (Fig. 3C), population sampling (Fig. 3D), or even misidentification (see also [35] for a comparable algorithm aimed specifically at trees with gene duplications only). The algorithm is built in to the user interface. For all single-locus trees with multiple terminals for at least one taxon, the user can retrieve either the original multree for further analysis, e.g., to distinguish paralogous from orthologous sequences, or the singly labeled tree, to obtain the maximum amount of species-level information contained in that particular tree.

*Multi-locus datasets*. Assembly of multi-locus concatenated data sets (“supermatrices”) is problematic when one or more of the data sets have multaxa [35]. We therefore used the reduced set of taxa obtained from the multree reduction as the source of sequence data for assembly of supermatrices. This results in a loss of some taxa on average (Fig. 3), but it also reduces the conflict within a gene tree arising from biological processes such as gene duplication and loss or incomplete lineage sorting. Although we have not built species trees using any methods aside from concatenation, our collection of reduced loci/trees could be used as inputs to species tree inference methods using consensus [41], reconciliation (e.g., [42, 43]) or explicit likelihood or Bayesian methods exploiting the sequence data proper (e.g., [21]).

Two protocols were used to guide selection of subsets of taxa and loci for assembly of multi-locus supermatrices from the single-locus reduced data sets in each NCBI hub group and all its descendant groups. Both generate multi-locus data sets with a desirable property, “decisiveness”, which can help limit the impact of missing entries in the supermatrix ([23, 24, 44–47]. A supermatrix, *M*, is decisive for tree, *T* (containing all taxa in *M*), if and only if the subtrees, t_i_, for each locus i, obtained by restricting *T* to only those taxa that have sequence data at locus i, uniquely define *T*. If, instead, the subtrees are consistent with more than one tree, they do not define *T*, and the supermatrix may be unable to distinguish between those trees for certain reconstruction methods (e.g., parsimony or partitioned likelihood analysis: [24]). A particularly strong form of decisiveness, which holds for some patterns of missing data, is that *M* may be decisive *for all possible trees*.

**Fig 4:**
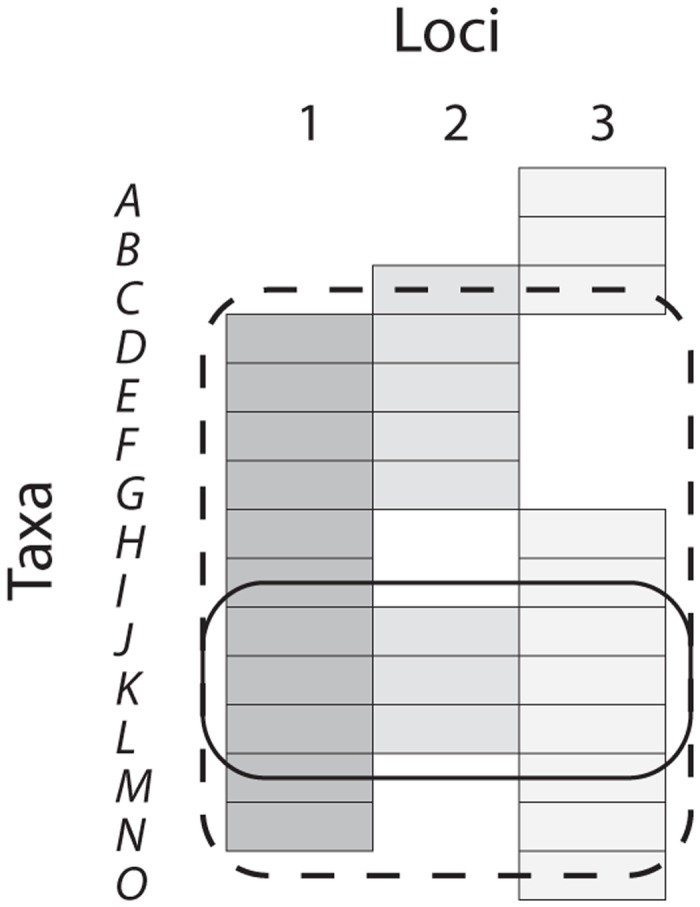
Data availability matrix. Filled bars indicate the presence of data for a particular locus and taxon. Multilocus datasets are constructed in two ways: (1) as bicliques (within the solid line), in which no taxa are missing any loci, and none can be added without introducing missing data (so they are “maximal”), and (2) as decisive quasi-bicliques (within the dashed line), in which a reference locus serves to restrict the taxon list, but all loci available for those taxa are included. Here locus 1 serves as the reference locus for the decisive quasi-biclique shown, but two others can be constructed by using the other two loci as reference loci. Similarly, other maximal bicliques are present, each containing only two loci (e.g., loci 2 and 3 for taxa C, J, K, L).

Our first protocol assembles maximal *complete* supermatrices by finding all so-called maximal bicliques in an associated graph data structure. Briefly, a biclique here refers to a set of taxa and loci for which all taxa have data for all loci; maximal bicliques can be found by exploiting graph theoretical results cited in [48, 49]. Since any supermatrix in which one locus includes sequence from all taxa is decisive, these are decisive for all trees. Our second protocol also guarantees decisiveness but allows some missing entries in the supermatrix. It builds a supermatrix using one locus as a reference locus. The taxon list is then restricted to those in the reference locus, but all available loci for each of those taxa are included (Fig. 4). Because of the reference locus, this supermatrix is also decisive for all trees, even though it contains missing data, and we refer to it as a *decisive quasi-biclique (dqbc)*. For a given collection of loci, one *dqbc* can be constructed using each locus as a reference in turn. Fig. 4 illustrates these kinds of data sets, including the trivially decisive case of single-locus data sets. In our implementation, we restricted the dqbc construction to include only those loci with at least 33.3% of the taxa in the reference locus.

The multi-locus datasets (maximal bicliques and decisive quasi-bicliques) built at some node in the NCBI hierarchy can and are expected to overlap with one another (Fig. 4). To ensure that the datasets are not entirely redundant with others constructed at the same NCBI node, various checks and filters were run on the results. We checked whether there were duplicate data sets within or between nodes in the NCBI hierarchy and whether any decisive quasi-bicliques were actually bicliques (which occurs rarely when the taxon coverage pattern is conducive). In addition we used a BLAST protocol to check that all loci in a data set are independent from each other, sharing no local homologies (these can arise occasionally for a variety of reasons upstream in the pipeline), which might lead to redundant inclusion in the same supermatrix (e.g., [12], corrigendum). The collection of multi-locus datasets can be large, in some cases with relatively dense taxon coverage, due to basic combinatorics. We found, for example, that within mammals there were hundreds of thousands of primate and carnivore bicliques (more than all the number of bicliques for all other taxa combined, in fact); we therefore sampled only a fraction of biqliques at random from these collections: 2% and 20% respectively.

The output of this pipeline is nearly one million “phylogenetically informative” data sets (i.e., having at least four taxa), among which 351,212 distinct taxa recognized by NCBI are distributed. For each data set, multiple sequence alignments using MUSCLE [50], ML optimal trees using default options in RAxML [51], and 1,000 “fast” parsimony bootstrap trees using PAUP* [52] were obtained. Computing time required is approximately 6 weeks on a 300 core linux cluster for the analyses described. We estimate that repeating this with full maximum likelihood bootstrap analyses with default options in RAxML (as opposed to the fast parsimony bootstraps used here) would require 5–50 years on the same hardware.

### The Database

*Schema, search and retrieval*. The STBase database has a very simple schema aimed at maximizing search and retrieval efficiency. Essentially it consists of five entities: taxa, sequences, clusters, data sets and confidence sets of trees. A taxon consists of a species or subspecific name and its NCBI taxon ID (both following NCBI’s taxonomy). A taxon can have multiple synonymous names mapped to the same taxon ID. Each sequence—represented by an NCBI GI number as its ID—is associated with a taxon, and there can be multiple sequences associated with the same taxon. A cluster is a collection of homologous sequences, loosely referred to as a “locus”. A data set is a collection of one or more aligned clusters/loci, concatenated into a supermatrix (if more than one), from which trees were constructed. Each data set is mapped to a set of one thousand bootstrapped trees. To map efficiently among these entities, STBase employs hash functions [53, 54] (string-specific: [55]), which are capable of inserting and deleting a random element in constant time irrespective of the size of the collection.

The user inputs a list of taxon names and/or genus names. Genus names are replaced by a list of all taxon names in that genus. This is followed by five steps: (1) retrieval of corresponding taxon IDs, (2) finding the data sets having the desired overlap with the set of query taxa and reading them from disk, (3) processing each data set to restrict each of its thousand trees to the taxa that overlap with the query, (4) summarizing the restricted trees for each cluster as a majority rule consensus tree, with support values, and returning these MRTs to the user. A similar approach is used on the website birdtree.org [14], which allows users to query sets of trees drawn from a pseudo-posterior distribution of complete bird trees constructed using a combination of data and simulation. Finally, (5) in the case of multrees, a singly-labeled reduced tree is computed on demand (this only applies to single-locus data sets—for multi-locus data sets, redundant sequences are handled prior to concatenation).

Because of the collective storage requirements of the trees (over 200GB), trees from all data sets cannot be kept in RAM, which poses several challenges to achieving fast query processing. Given a set of taxon IDs, identifying overlapping clusters and reading them from disk memory is the most time consuming part of the query process, as there are nearly one million data sets, with 4 to nearly 10,000 taxa each, covering more than 340,000 taxa (Table 1). However, STBase identifies overlapping clusters in time that is independent of the size of the database by using inverted indexing [56, 57]. An inverted index enables search and retrieval of a subset of “documents” (here data sets) containing one or more words from the query set. It does so by maintaining a mapping from a predefined set of keywords to the documents in the collection that contain them. In STBase, the goal is to find the data sets containing taxa that map to the list of taxa supplied by the user. STBase’s inverted index therefore stores exactly *which* data sets (“documents”) contain taxon names (“keywords”) and *where* those data sets are located on the hard drive.

*Majority rule tree generation*. A query typically finds 100–200 data sets having sufficient overlap with the taxon names provided as input. Each of these is associated with a thousand pre-computed bootstrapped trees that are each restricted to the query overlap. These 1000 pruned trees are then summarized as an MRT. To generate the MRT at query time (“on-the-fly”), we used Amenta et al.’s [58] randomized linear time MRT algorithm, which uses hash codes—a constant size object—to represent bipartitions and a clever method to construct the MRT using only these hashed bipartitions. This results in an expected linear-time (i.e., optimal) algorithm.

## Utility

### User Interface

**Fig 5:**
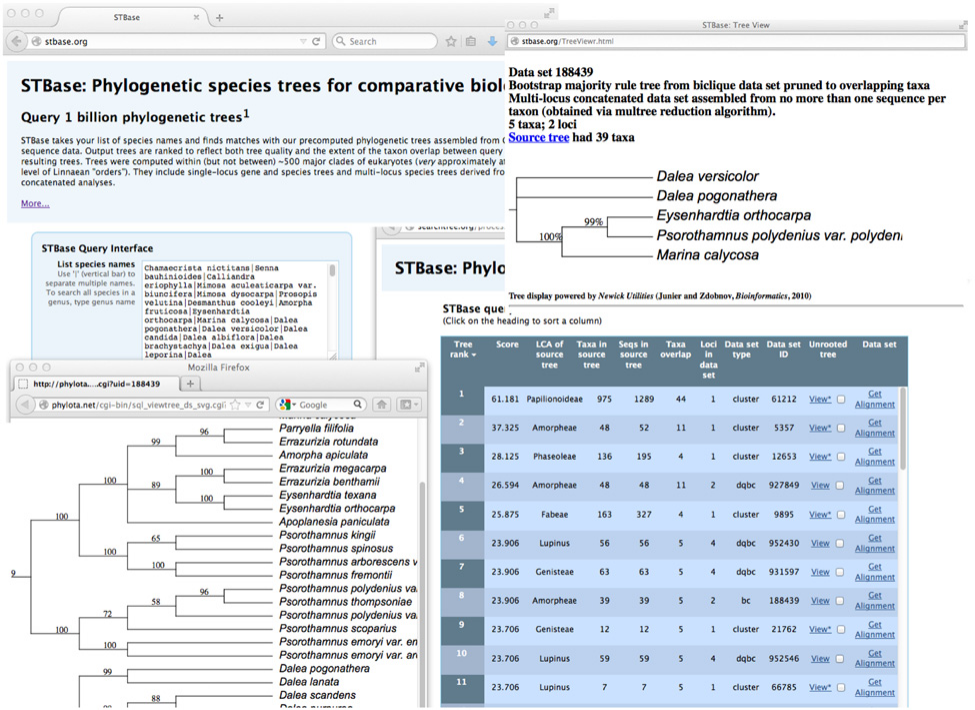
Screenshot of the STBase user interface. Query taxa are shown in the query box at upper left. Top hits are ranked in the list at lower right. One of these is selected for viewing at upper right. Lower left tree shows the larger source tree from which the overlapping subtree was extracted.

Fig. 5 shows a screenshot of the user interface. The user can enter a list of up to 10,000 species or subspecific taxon names as multinomials, following the NCBI taxonomy. Any uninomial is assumed to represent a genus name, and all species in that genus are added to the query. On the search page the user can optionally increase the required minimum taxonomic overlap to reduce the number of trees returned and can also select the format of taxon names for subsequent download after retrieval.

The output consists of a simple table layout of ranked hits, each row corresponding to a data set and its confidence set of trees. To orient the user to the phylogenetic scope of the trees returned, the last common ancestor (LCA; also known as “most recent common ancestor”) of the data set (within the NCBI hierarchy) is computed and returned (using an efficient LCA implementation: c.f. [27]). The weighting parameter for overlap vs. tree quality can be adjusted on this page with a slider bar, which instantly re-orders the retrieved trees. A variety of data sets can be accessed from this page as well, including a nexus formatted multiple sequence alignment, a nexus formatted tree file for the overlapping subtree, and the multree reduction of any single cluster trees for which this has been computed. Metadata for the loci included in multi-locus data sets are embedded in the sequence alignment file. Single-locus trees are rooted (provisionally and no doubt approximately) by reference to a midpoint-rooted [59] optimal ML tree of the entire source tree, which is constructed and stored elsewhere in the database.

### Use Case: Genome Size Variation in Cactaceae

**Fig 6:**
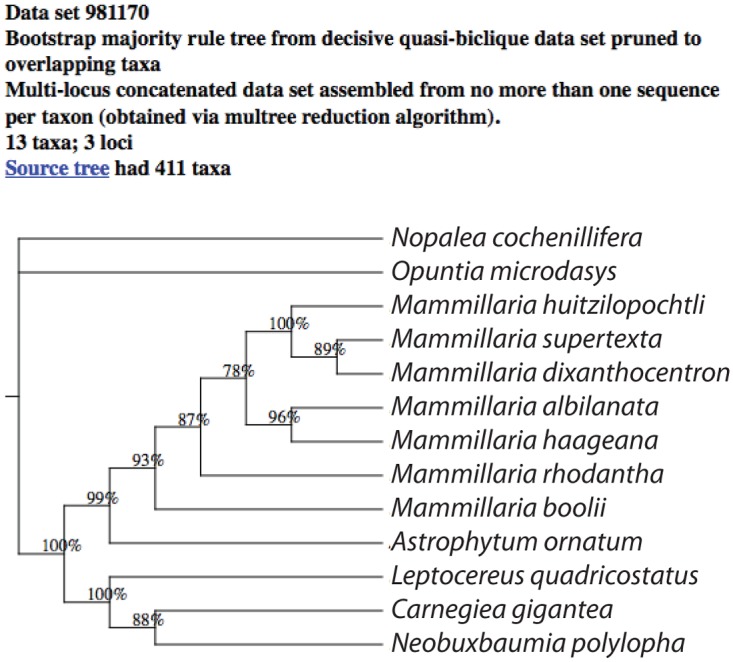
Tree view. Highest ranking tree resulting from query on 50 species names in Cactaceae for which genome sizes are available.

We illustrate a typical “use case” for the database by the following example. As a prelude to genomic investigation of the angiosperm family Cactaceae (the cacti), a user would like to understand the variation in genome size across the family and undertake ancestral state reconstruction to see if some groups’ genomes are evolutionarily more labile than others. The Kew Plant DNA C-value database (http://data.kew.org/cvalues/; release 6.0) is queried and a list of all 50 species (in 21 genera) is obtained for which C-values are known. The 50 names are submitted to STBase and 89 resulting hits are obtained in 1.5 seconds, with scores ranging from 19–69.9. The best scoring result (using the default value *h* = 1.0) is a tree containing 14 of the original 50 taxa (from 7 of 21 genera), and is based on a 3-locus decisive quasi-biclique data set. Average bootstrap values are quite high in the tree (Fig. 6). For context the original source tree, having 411 taxa, can be viewed via a hyperlink from the tree display page. Average bootstrap values across this much larger tree are less, which illustrates the power of our subtree pruning to extract an assessment of strong signal for the relationships most germane to the taxa for the available comparative data. A tree file with all results and a table with summary statistics can be downloaded. The complete supermatrix alignment can also be downloaded directly from the results page, which includes the original GenBank GI numbers of all sequences. If desired, the user is then free to rebuild the tree using more computationally intensive inference methods (the alignment can also be redone). At this point the user could perform comparative analyses on the C-value data, such as ancestral state reconstruction.

Optionally, the user might increase the taxonomic overlap by running the search again at the genus level. Our implementation replaces a submitted genus name with all the species in that genus that are currently in the database, and then runs the query as usual. The user at that point might be willing to use that species as a proxy for the actual species in the same genus for which the *C*-value data are available. In our use case, when the 21 genus names are submitted to the query engine, 508 resulting trees are obtained in 3.1 seconds, with scores ranging from 0.8 to 43.1. The best scoring result is a 4-locus decisive quasi-biclique data set with 162 taxa from 11 of the 21 genera. It comes from an original larger tree of 392 taxa. The best scoring tree returns *all* species from the original source tree in each of the overlapping genera, so, for example, most of the species in the tree are in *Mammillaria*, many more than are present in the genome size data set. It is up to the user to decide which proxy species may be appropriate to map the *C*-value data from a species to a congener in this tree. Overall the bootstrap support values are fairly good, although there is lack of resolution within *Mammillaria*.

### Efficiency

As a result of techniques described above and some careful preprocessing of the data, STBase answers queries in time that is linear in the total size of the query plus the output, and independent of the size of the underlying tree repository. Retrieval times for queries of ~50 names on our database of 1 million data sets and 1 billion trees typically require 5–15 seconds. However, because the time is linear in the size of the output, query time can be significantly longer when the number of hits is very large. For example, a query on the genus name *Felis* (alone) or *Drosophila* (alone) finds a very large number of hits that must be retrieved, ranked and processed. The search engine by default limits output to 1,000 records, each computed from the first 100 bootstrap replicates only. For queries returning longer lists, this is not guaranteed to return an optimal ranking, and modifying the defaults is a good idea. In the future we want to explore a “seeding” strategy, in which particularly poor candidates are immediately filtered out by computation on a very small number of bootstrap replicates.

## Discussion

### Tree quality

Our pipeline was designed to limit upstream errors due to multiple sequence alignment problems in highly divergent taxa by restricting data set assembly to occur within but not between 500+ “hub groups” of eukaryotes. Within these groups, we used “fast” parsimony heuristics to build large confidence sets of tree. Although these tend to produce conservative tree estimates with bootstrap scores lower than those using more exhaustive heuristics [60], the quality of trees was quite good on average. Table 1 reports the average fraction of nodes resolved in the bootstrap MRT, which is an aggregate indication of tree quality. Efforts to engineer decisive multi-locus data sets may explain the higher values in those data sets, but presumably these values are also due to presence of multiple loci and the smaller average size of trees, which is correlated with increasing bootstrap proportions [39]. Our first release of STBase also relies on fast parsimony algorithms for computational reasons. The 5–50 years of computing that would currently be required for full (not fast) ML runs is obviously out of bounds. Because our aim is to provide quality assessments for user-generated subtrees (based on the taxa of interest), branch-based probabilistic approaches such as the approximate likelihood ratio test [61] cannot be used here.

### Rationale for Data Set Assembly Strategy

The data sets in STBase overlap with one another. In mathematical terms, they represent a “cover” of the underlying sequence data, rather than a “partition” of it. Each data set in STBase comprises a different collection of sequences, taxa and loci, but these collections can overlap partially with each other. The effect of this is somewhat analogous to coverage in genome sequence assembly, where multiple reads allow evaluation of mistakes, except that in phylogenetic inference the mistakes(s) can arise for many inferential reasons. For example, suppose we are interested in a set of taxa, *U*, common to two different data sets, having taxon sets *X*_1_ and *X*_2_: thus *U* = *X*_1_ ∩ *X*_2_. After building alignments and trees from *X*_1_ and *X*_2_ separately, we might well discover that the subalignments and subtrees corresponding to just our taxa of interest, *U*, are different for any number of reasons. Both the optimal alignment and optimal tree given the alignment for the taxa in U can depend on the context, that is, the other taxa in *X*_1_ or *X*_2_. This is part and parcel of the longstanding debate over adding taxa vs. loci in phylogenetic analysis [62]. By assembling data sets with many different contexts, including different phylogenetic scales (levels in the NCBI hierarchy), different numbers of loci, and different patterns of missing data (bicliques vs. decisive quasi-bicliques), we hope the database exposes sensitivity to these factors. By listing these different data sets ranked by quality in the output, the interface naturally encourages exploration of these effects, and we would encourage users to consider, for example, studying alternative alignments of the largest clusters. The important caveat emptor is that users should not be tempted to take multiple data sets returned in a search and perform subsequent phylogenetic or statistical analyses on them assuming they are statistically independent. Any one data set, however, reflects a non-redundant sample of sequence data.

### Species Trees and Gene Tree Conflict

One hallmark of STBase is that it archives estimates of species trees. More precisely it reports singly-labeled trees in which labels correspond to NCBI taxa at the lowest rank to which they have been identified. We do this, not optimally, but conservatively. In other words, each multree from a single-locus data set is reduced to a singly labeled subtree in such a way that it does not introduce any conflict with the original tree [37]. This often entails loss of resolution and/or loss of taxa, but the taxon loss is less than it would be if all duplicate taxa were removed, an admittedly naïve alternative [37]. These reduced sets of taxa then form the basis of multi-locus data set assembly. This multree reduction algorithm is not optimal because it does not exploit all of the information present in the original multrees, some of which (such as numbers of gene duplications, or deep coalescence events) can be helpful in inferring a more complete species tree [63]. However, exploiting this information requires making assumptions about the sources of multaxa in the data sets, something we cannot glean uniformly from GenBank annotations or the raw sequence data themselves. The structure of the database can accommodate other methods of assembling species trees, but at the moment the number of alternative methods is quite large, and we leave this to future work.

### Applications in Comparative Biology and Large Tree Construction

Many, though not all, questions in comparative biology have a phylogenetic scope limited to major clades, and can thus be addressed by the trees within hub groups in STBase. Many problems in comparative physiology, functional morphology, developmental biology and comparative genomics are largely within the scope of species in the same taxonomic genus, family or order. On the other hand, STBase does not currently contain trees that span between our 500+ hub groups. Therefore a query list sampled across all plants in a community or regional flora (cf. [15]), for example, would return a set of trees, each of which is confined to a major clade of plants. A user with such a query would then be compelled to pursue their own supermatrix or supertree analyses to combine these results into a more comprehensive tree.

We were reluctant to transcend the scope of our hub groups for several reasons. The problems of scaling data set assembly, multiple sequence alignment, and tree inference using nucleotide sequences beyond thousands to 10s of thousands of taxa are daunting [12]. Moreover, we suspect that the exploitation of a small number of idiosyncratic high quality scaffold data sets will be necessary to tie together trees between major groups [14]. For example, Soltis et al.’s [45] analysis of 17 loci for 640 angiosperms was a decisive multi-locus data set that could form the scaffold for our smaller trees among angiosperms, as could others. However, how these data should be incorporated with a large collection of smaller data sets is unclear, and no doubt raises many issues about supertree vs. supermatrix construction, as well as the proper handling of missing data.

### Big data

Given the combination of vast sequence data resources and computationally intractable inference problems, few would doubt the assertion that phylogenetics is “big data” science. A few observations gleaned in the construction of STBase provide some support for this notion. First, it became clear that storage of an entire confidence set of trees rather than a single tree (or a few alternative optimal trees) let us build a tool for exploration of the statistical support for phylogenetic hypotheses tailored to the user’s taxon list, in real time. However, a consequence of this is the need to store 2–3 orders of magnitude more phylogenetic trees in the database. Although compression of these trees is possible [64, 65], there will be a tradeoff between decompression speed and savings in database storage. Second, we selected only a small number of protocols for assembling data sets for tree construction. These were guided by some theoretical results on the impact of missing data in multi-locus data sets. Many other protocols could be designed to emphasize different aspects of data set structure, such as ones taking note of other measures of information content [66], or to exploit the many different available species tree inference methods [34]. Given sufficient computing resources, the number of data sets might easily be increased by 1–2 orders of magnitude by including such protocols. Finally, the 6 million sequences used to build STBase represent only a few percent of GenBank, the “taxonomically enriched” part, largely neglecting the vast quantities of high throughput sequence data that are available (still) for a relatively limited number of taxa. Including these data would scale up analysis in our pipeline by two orders of magnitude, although perhaps not the number or size of trees to the same degree. However, if metagenomic data sets were ultimately included, the size of something like STBase might well approach 10^13^–10^15^ trees. Databases of that size or larger exist now (e.g., NCBI’s Sequence Read Archive, or Shutterfly’s image database: [67]), but tailoring database tools to handle tree collections of this size while allowing efficient tree-based queries and other operations specific to phylogenetic analysis may well require new algorithms and algorithm engineering.
